# Proactive Cystoscopic Debris Removal for Reducing Catheter Blockage in Patients with Long-Term Indwelling Catheters: A Prospective Self-Selected Cohort Study with Exploratory Subgroup Analysis on Urinary Tract Infections

**DOI:** 10.3390/jcm15135217

**Published:** 2026-07-03

**Authors:** Meng-Hsuan Lu, Yu-Hui Huang, Yun-Sheng Chen, Kai-Siang Chen, Chieh-Jui Chen, Sung-Lang Chen

**Affiliations:** 1 Department of Urology, Chung Shan Medical University Hospital, Taichung 402, Taiwan; kye961220@gmail.com; 2 School of Medicine, Chung Shan Medical University, Taichung 402, Taiwan; yhhuang59@hotmail.com; 3 Department of Physical Medicine and Rehabilitation, Chung Shan Medical University Hospital, Taichung 402, Taiwan; 4 Department of Obstetrics and Gynecology, Changhua Christian Hospital, Changhua 500, Taiwan; gracelucky028@gmail.com; 5 The Center of Humanities and Society, Chia Nan University of Pharmacy &Science, Tainan 717, Taiwan; cks888@mail.cnu.edu.tw; 6 Graduate Institute of Business Administration, Fu Jen Catholic University, New Taipei City 242, Taiwan; 7 School of Medicine, College of Medicine, Taipei Medical University, Taipei 110, Taiwan; chenjerry123@gmail.com

**Keywords:** CAUTI, bladder debris, cystoscopy, spinal cord injury, neurogenic bladder, long-term catheterization

## Abstract

**Background**: Catheter-associated urinary tract infections (CAUTIs) are a major concern in patients with long-term indwelling urinary catheters, especially those with neurogenic bladder. This study assessed whether proactive cystoscopic removal of bladder debris reduces symptomatic UTI incidence and catheter blockage. **Methods**: This prospective, self-selected cohort study was conducted between January 2022 and December 2025 at a tertiary center in Taiwan. Enrollment occurred from January 2022 to June 2024, with follow-up completed by December 2025. Patients chose standard CDC-guided care (control, *n* = 63) or standard care plus flexible cystoscopy every 3 months for gentle low-volume evacuation (<100 mL normal saline) of dependent bladder debris (intervention, *n* = 141). Inverse probability of treatment weighting (IPTW) was used to address selection bias. Symptomatic UTIs were prospectively recorded using strict criteria. Cumulative incidence was analyzed with Kaplan–Meier methods and multivariable Cox regression. **Results**: After IPTW, baseline characteristics were well balanced. Median follow-up was 26 months (IQR 18–34). The incidence of catheter blockage was significantly lower in the intervention group (7.8% vs. 22.2%, *p* = 0.004). In the overall cohort, the reduction in symptomatic UTI incidence did not reach statistical significance (49.2% vs. 36.9%, *p* = 0.092). In the pre-specified spinal cord injury (SCI) subgroup (*n* = 71), the intervention was associated with improved UTI-free survival (log-rank *p* = 0.03; adjusted HR 0.52, 95% CI 0.28–0.96, *p* = 0.037; treatment × SCI interaction *p* = 0.042). All adverse events were Clavien–Dindo Grade I. No major complications occurred. **Conclusions**: Proactive gentle cystoscopic debris removal was associated with reduced catheter blockage. A signal toward lower symptomatic UTI risk was observed in the SCI subgroup, but not in the overall cohort. Due to the self-selected design and residual confounding, these findings are hypothesis-generating.

## 1. Introduction

Catheter-associated urinary tract infections (CAUTIs) represent one of the most common healthcare-associated infections worldwide and impose a substantial clinical, economic, and humanistic burden [1,2]. Long-term indwelling urinary catheters are often necessary for patients who are unable or unwilling to perform intermittent catheterization [3,4], particularly those with neurogenic bladder dysfunction due to spinal cord injury (SCI), stroke, multiple sclerosis, or advanced frailty. In these patients, CAUTIs occur frequently, often leading to recurrent hospitalizations, emergency visits, urosepsis, catheter blockages, bladder stone formation, and markedly reduced quality of life. In Taiwan and many developed healthcare systems, patients with long-term catheterization face annual symptomatic UTI rates ranging from 30 to 70% [5,6,7], with spinal cord injury patients being at particularly high risk due to impaired bladder sensation, detrusor areflexia, chronic urinary stasis, and inability to empty the bladder effectively [8].

The pathogenesis of CAUTI in this population is closely linked to the accumulation of bladder debris, including mucus, crystals, clots, and bacterial biofilm, which tends to sediment in the dependent portion of the bladder [9,10]. Standard catheter care and conventional irrigation techniques frequently fail to effectively clear this dependent debris because the drainage eyes of Foley catheters do not reach the bladder fundus, and bladder wall collapse during aspiration further limits efficacy [11]. This persistent debris serves as a nidus for biofilm formation, bacterial colonization, and recurrent infection.

According to the European Association of Urology (EAU) guidelines on Urological Infections, treatment of asymptomatic bacteriuria—defined as cloudy or foul-smelling urine without other symptoms—provides no clinical benefit in patients with indwelling catheters [12]. Despite this, clinicians frequently misclassify asymptomatic bacteriuria as symptomatic UTI, leading to inappropriate antibiotic use. Such overuse accelerates the development of antibiotic resistance. Supporting this concern, a recent study reported that pathogens responsible for CAUTIs exhibit significantly higher resistance rates to commonly used empirical antibiotics compared with non-catheter-related pathogens [13,14]. The implications are serious: rising antimicrobial resistance narrows therapeutic options for complicated infections, prolongs illness, escalates healthcare costs, increases mortality risk, and contributes to the global crisis of multidrug-resistant organisms [15].

This study was motivated by repeated clinical observations in our tertiary neuro-urology practice, where many patients with unavoidable long-term indwelling catheters continued to experience frequent catheter blockages and symptomatic UTIs despite adherence to standard Centers for Disease Control and Prevention (CDC) -guided care. We hypothesized that proactive, periodic, gentle cystoscopic removal of dependent bladder debris under direct vision, using minimal saline volume (<100 mL), could more effectively interrupt the cycle of debris accumulation, biofilm formation, and recurrent infection compared with conventional approaches, while minimizing additional antibiotic exposure.

Therefore, we conducted this prospective, self-selected cohort study to evaluate the efficacy and safety of scheduled flexible cystoscopic debris removal as an adjunct to standard care in patients with long-term indwelling catheters, with particular interest in the high-risk SCI population.

## 2. Materials and Methods

### 2.1. Study Design and Participants

This prospective, self-selected cohort study was conducted at Chung Shan Medical University Hospital, Taichung, Taiwan, between January 2022 and December 2025. Because patients were enrolled consecutively between January 2022 and June 2024 and followed until December 2025, individual follow-up durations ranged from a minimum of 18 months (for the latest enrollees) to a maximum of 48 months (for the earliest enrollees), resulting in a cohort median follow-up of 26 months (IQR 18–34). This study was conducted in accordance with the Declaration of Helsinki and approved by the Institutional Review Board of Chung Shan Medical University Hospital (approval number: CS2-25145). Informed consent was obtained from all participants prior to enrollment.

This study was reported according to the STROBE guidelines for observational studies. 

Adult patients aged ≥18 years who had undergone indwelling urinary catheterization for ≥6 months were eligible. The SCI subgroup analysis was pre-specified in the study protocol. Exclusion criteria included active UTI on enrollment, recent bladder surgery or tumor (<3 months), coagulopathy, or therapeutic anticoagulation. Active UTI at the time of screening was defined as a positive urine culture (≥10^5^ CFU/mL) accompanied by acute systemic symptoms (fever > 38 °C) or acute localized urological changes requiring active antibiotic therapy.

After detailed counseling, patients self-selected into standard CDC-guided catheter care (control group, *n* = 63) or standard care plus scheduled flexible cystoscopy every 3 months for debris removal (intervention group, *n* = 141).

### 2.2. Intervention

Flexible cystoscopy was performed every 3 months in an outpatient setting by experienced urologists. Dependent debris was gently evacuated with <100 mL normal saline irrigation and aspiration. No routine prophylactic antibiotics were used in either group. Antibiotics were prescribed only for episodes meeting the study’s strict symptomatic UTI criteria. The control group received standard CDC-recommended catheter care, including as-needed irrigation when indicated. Maintenance of a strictly closed drainage system, daily meatal-catheter junction cleansing with plain soap and water, twice-daily drainage bag evacuation, and routine catheter exchanges every 2 to 4 weeks depending on the material (silicone vs. latex). Routine bladder irrigation is not performed in either arm; manual irrigation is permitted in the control group solely as an as-needed rescue maneuver using a 50 mL catheter syringe with normal saline only when mechanical lumen occlusion by thick sludge or clots is clinically suspected.

To ensure high procedural consistency and safety during the 3-month intervention cycles, strict quality assurance measures were implemented. All flexible cystoscopic evacuations were performed exclusively by three dedicated senior urologists who underwent standardized training on the low-volume aspiration protocol prior to trial initiation. Additionally, random internal procedural audits were conducted bi-annually by the principal investigator to verify strict adherence to the fluid volume limits (<100 mL) and vacuum pressure rules.

### 2.3. Outcome Measures

UTI events were prospectively adjudicated by two independent research nurses using standardized case report forms, with final confirmation by the principal investigator. Although complete blinding was not feasible, outcome assessors were not directly involved in the cystoscopic procedures.

UTI events were recorded through chart review and patient interviews by research staff. To minimize misclassification, strict definitions aligned with the National Healthcare Safety Network (NHSN) criteria were used:

Major UTIs were operationally defined as episodes requiring acute hospitalization, intravenous antibiotic therapy, or presenting with documented systemic hyperpyrexia (>38 °C).

Minor UTIs required localized changes (new-onset suprapubic pain, worsening incontinence, or acute autonomic dysreflexia spikes in SCI patients) accompanied by cloudy or foul-smelling urine, or autonomic dysreflexia in patients with SCI, resulting in prescription of targeted oral antibiotics. Asymptomatic bacteriuria was excluded.

The primary outcome was cumulative incidence of symptomatic UTIs. Secondary outcomes included catheter blockage requiring intervention and adverse events graded by Clavien–Dindo classification.

UTI events were prospectively adjudicated by two independent research nurses using standardized case report forms, with final confirmation by the principal investigator. Although complete blinding was not feasible, outcome assessors were not directly involved in the cystoscopic procedures. Inter-rater reliability for major UTI classification was high (Cohen’s kappa = 0.87) and moderate for minor UTI classification (Cohen’s kappa = 0.68).

Symptomatic UTI events were verified through a hybrid methodology comprising retrospective chart reviews cross-referenced with a standardized clinical symptom telephone checklist executed during scheduled telephone follow-ups. Mechanistically, our laboratory infrastructure was restricted to routine clinical microbiology pipelines. No physical matrix staining, scanning electron microscopy, or molecular sequencing assays were performed on the aspirated debris or catheters to measure true biofilm composition. This represents a clear diagnostic limitation, as conventional cultures are blind to non-planktonic, viable but non-culturable (VBNC) organisms embedded deep inside the slime layer.

### 2.4. Statistical Analysis

To address selection bias, inverse probability of treatment weighting (IPTW) with stabilized weights was applied. Covariate balance was assessed using standardized mean differences (SMD) before and after weighting. Stabilized weights had a mean of 1.02 (range 0.45–2.81). Sample size was determined based on pilot data, aiming to detect a 40% relative reduction in symptomatic UTI rate with 80% power at α = 0.05, leading to the target enrollment of approximately 200 patients. Crude proportions are presented for clinical interpretability, while Kaplan–Meier curves and Cox regression models were performed on IPTW-weighted data.

The subgroup analysis for patients with spinal cord injury (SCI) was prospectively pre-specified during institutional protocol development due to their high clinical risk of neurogenic stasis. However, because this was an observational study, formal statistical adjustments for multiplicity (e.g., Bonferroni correction) were not applied to the subgroup analyses, and these results must be interpreted as exploratory findings.

Cumulative UTI-free survival was estimated using Kaplan–Meier curves with log-rank tests. Multivariable Cox proportional hazards models adjusted for age, sex, diabetes, catheter type, and prior-year UTI frequency. The proportional hazards assumption was verified using Schoenfeld residuals. The global test was not significant (*p* = 0.78), and visual inspection of residual plots showed no obvious violations of the assumption for the primary exposure or major covariates. (*p* > 0.05 for all covariates). Patients lost to follow-up were right-censored at their last visit. Analyses were performed using SPSS 26.0 and R 4.3.0. A two-sided *p* < 0.05 was considered significant.

## 3. Results

### 3.1. Baseline Characteristics and Follow-Up

**Figure 1:**
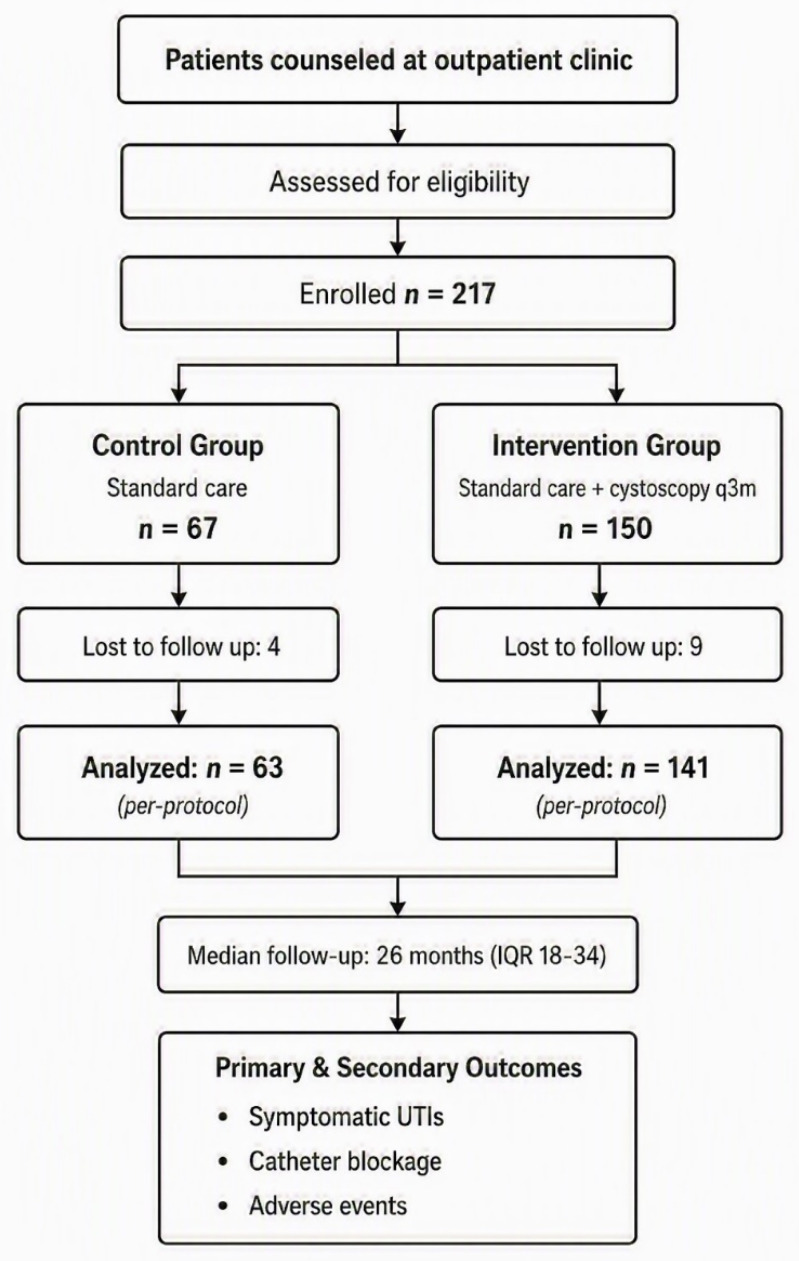
Study flow diagram outlining screening, assignment, and final sample size retention.

**Figure 2:**
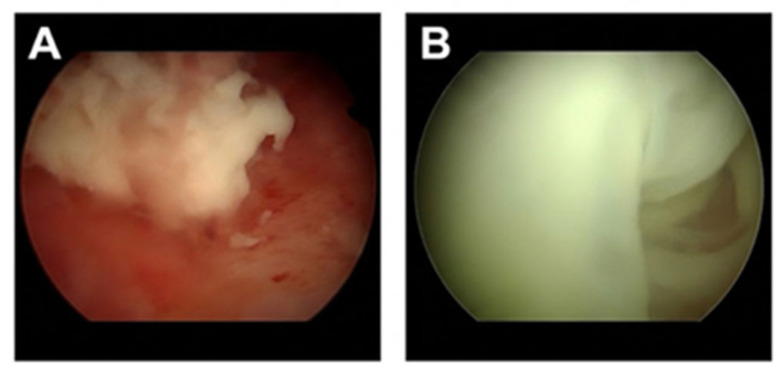
Cystoscopic appearance of bladder debris and biofilm in patients with long-term indwelling catheters. (**A**) Dense, white, amorphous encrustation and fluffy debris floating and adhered to inflamed bladder mucosa. (**B**) Panoramic view showing extensive, continuous, smooth yellowish-white biofilm layer covering the bladder mucosa. These images illustrate the dependent debris and biofilm burden that the intervention aimed to address.

A total of 217 patients were initially enrolled (67 in the control group and 150 in the intervention group). Thirteen patients (6.4%) were lost to follow-up (4 in the control group and 9 in the intervention group), primarily due to relocation or transition to palliative care. The final analyzed cohort was 204 (63 in the control group and 141 in the intervention group). Median follow-up duration was 26 months (interquartile range 18–34 months), with no significant difference between the two arms (*p* = 0.41). Figure 1 shows the study flow diagram. Representative cystoscopic images of bladder debris and biofilm are presented in Figure 2.

Adherence to the 3-month flexible cystoscopy schedule was high within the intervention arm (91.2% of sessions occurred within the protocol window). A total of 12 patients experienced minor scheduling deviations due to personal scheduling conflicts or intercurrent non-urological illnesses, but all returned for their scheduled procedure within an extended 4-week window and were retained in the final analysis.

After IPTW, baseline characteristics, including the frequency of UTIs in the 12 months prior to enrollment, were well balanced between groups, with all standardized mean differences (SMD) < 0.10 (Table 1).

### 3.2. Catheter Blockage and Safety Outcomes

Catheter blockage occurred in 7.8% of the intervention group versus 22.2% of the control group (*p* = 0.004). In the overall cohort, the reduction in symptomatic UTI incidence did not reach statistical significance (49.2% vs. 36.9%, *p* = 0.092). (Table 2). All procedure-related adverse events were Clavien–Dindo Grade I (mild hematuria 4.3%, transient low-grade fever 2.8%, minor discomfort 5.0%). No Grade II or higher adverse events (Table 3).

### 3.3. UTI Outcomes

**Figure 3:**
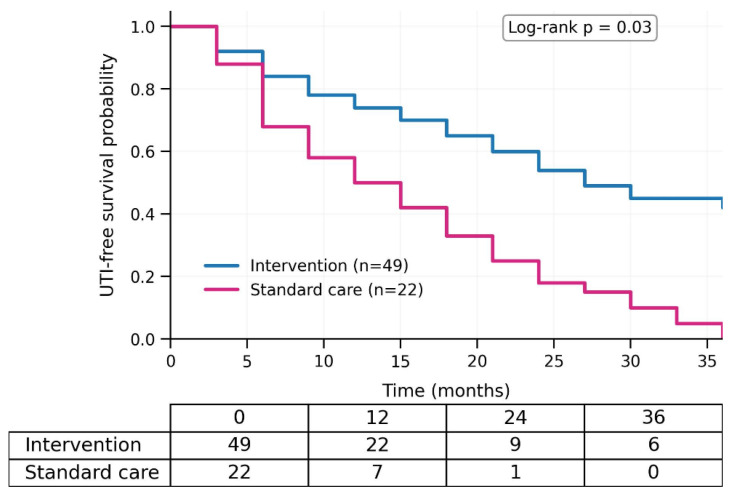
Kaplan–Meier estimates of cumulative symptomatic urinary tract infection (UTI)-free survival probability in the spinal cord injury (SCI) subgroup (*n* = 71).

In the overall cohort, the intervention was associated with a non-significant reduction in incidence of symptomatic UTI. In the pre-specified spinal cord injury (SCI) subgroup (*n* = 71), Kaplan–Meier analysis demonstrated significantly better cumulative UTI-free survival in the intervention arm (log-rank *p* = 0.03) (Figure 3 and Table 4). A formal test for treatment-by-SCI interaction was not statistically significant (*p* = 0.12). Multivariable Cox regression confirmed the intervention as an independent protective factor (adjusted HR 0.52, 95% CI 0.28–0.96, *p* = 0.037) (Table 5).

A formal test for interaction between treatment allocation and SCI status was statistically significant (*p* = 0.042), indicating that the clinical benefit of cystoscopic debris removal on reducing symptomatic UTIs was significantly more pronounced in the neurogenic bladder milieu of SCI patients than in non-SCI patients.

Urine culture results were available for 119 symptomatic UTI episodes (78.3%). The positivity rate, pathogen distribution, and proportion of multidrug-resistant organisms were similar between the two groups (Table 6), suggesting that the reduction in UTI events in the intervention arm was not accompanied by a shift in microbiology.

Supplementary Table S1 Multivariable Cox Proportional Hazards Model Diagnostics and Covariate Parameters (SCI Subgroup, *n* = 71)

Supplementary Table S2: Details of survival analysis—events, censoring, median time to first UTI, etc.

## 4. Discussion

CAUTIs continue to represent a major clinical challenge in patients requiring long-term indwelling urinary catheters. This prospective self-selected cohort study suggests that proactive cystoscopic removal of bladder debris using a gentle, low-volume technique (<100 mL normal saline) is feasible and may be associated with a reduction in catheter blockage and symptomatic UTI risk, with the most notable signal observed in the high-risk SCI population.

Compared with previously reported bladder debris management strategies, our approach offers several distinct advantages. A plausible biological rationale exists for this intervention. In patients with long-term catheterization, particularly those with neurogenic bladder dysfunction, debris (including crystals, clots, mucus, and biofilm) tends to sediment gravitationally into the most dependent portion of the bladder due to impaired detrusor contractility and urinary stasis. Conventional catheter irrigation is frequently ineffective because the drainage eyes of standard Foley catheters do not reliably reach the dependent fundus, and irrigation fluid often fails to dislodge or aspirate settled material [16,17]. High-volume bladder irrigation or pulsatile systems have been attempted, yet they carry risks of urothelial trauma, bacterial dissemination, and discomfort. Other techniques, such as antibiotic-impregnated catheters or bladder instillations (e.g., with gentamicin or taurolidine), primarily target bacterial load rather than the underlying debris scaffold and raise concerns regarding antimicrobial resistance and long-term safety [18,19]. Bladder wall collapse during aspiration further limits efficacy, while biofilm-embedded debris resists simple saline flushing. These anatomical and technical limitations help explain the mixed results of previous irrigation studies and the cautionary stance of major guidelines, including those from the CDC [20,21].

Although a patented weighted catheter design aims to improve dependent drainage by allowing the distal tip to settle gravitationally [22,23], it still lacks the precision of direct visualization and controlled mechanical removal. In contrast, our cystoscopic approach allows direct visualization and targeted gentle evacuation of debris and biofilm with minimal saline volume. By avoiding aggressive or high-volume irrigation, this technique minimizes the risk of bladder distension, urothelial disruption, or bacterial dissemination that could paradoxically increase infection risk. The similar microbiological profiles between groups further support that the observed clinical benefit is primarily attributable to debris removal rather than selection of different bacterial strains.

The stability of the baseline microbiological profile post-intervention is a significant clinical finding. Proactive flexible cystoscopy focuses purely on the mechanical evacuation of the sedimented debris scaffold from the dependent bladder fundus under direct vision. Unlike continuous antibiotic prophylaxis, this mechanical approach exerts no selective biochemical pressure on local bacteria. Consequently, it may reduce the intravesical bacterial burden and potential infection foci without promoting the emergence of multidrug-resistant organisms (MDROs). Furthermore, our strict adherence to a gentle, low-volume, low-pressure irrigation technique (<100 mL normal saline) avoids fluid-shear micro-trauma, protecting the bladder’s defensive glycosaminoglycan layer and preventing opportunistic pathogen colonization. However, standard clinical urine cultures only capture dominant, planktonic aerobic uropathogens (≥10^5^ CFU/mL). They are inherently unable to detect subclinical ecological rearrangements within complex biofilm architectures or fastidious, anaerobic strains, which warrant future metagenomic sequencing evaluation.

Interestingly, while per-patient UTI incidence showed only borderline reduction in the overall cohort, the number of UTI episodes per patient was significantly lower in the intervention arm (median 0 vs. 1, *p* = 0.035) (Table 2) suggesting a possible effect on recurrence rather than first occurrence. These finding warrants confirmation in larger trials. The most promising finding was observed in the SCI subgroup, where the intervention was associated with improved cumulative UTI-free survival (log-rank *p* = 0.03) and a reduced hazard of symptomatic UTI after multivariable adjustment (adjusted HR 0.52, 95% CI 0.28–0.96). In contrast, the reduction in symptomatic UTI in the overall cohort was smaller and did not reach statistical significance. This benefit is biologically plausible given the profound detrusor areflexia, poor bladder sensation, and chronic urinary stasis characteristic of neurogenic bladder [24]. In such patients, the dependent bladder becomes a stagnant reservoir where debris serves as a scaffold for biofilm formation and creates an ideal environment for recurrent infection [25]. The intervention is hypothesized to physically disrupt the sedimented debris matrix, which may act as a potential nidus for biofilm propagation, though this associative relationship requires prospective direct quantification to verify. Conversely, non-SCI patients (e.g., those with stroke, neurodegenerative diseases, or frailty) often retain some degree of bladder sensation or partial detrusor function, leading to less severe stasis and a lower baseline debris burden. Consequently, the incremental benefit of proactive cystoscopic intervention was more modest and less statistically detectable in the overall heterogeneous cohort. Furthermore, no prophylactic antibiotics were used in either the intervention or control group. This study design eliminates antibiotic prophylaxis as a potential confounder and suggests that the observed benefit is primarily due to regular mechanical removal of bladder debris rather than antimicrobial effects.

Nevertheless, the results must be interpreted with considerable caution due to the non-randomized, unblinded, self-selected design of this cohort study. This introduces inherent selection bias by indication, as patients with a heavier baseline symptom burden, more frequent catheter blockages, or severe debris accumulation may have been systematically more motivated to choose the intervention arm. While statistical techniques like inverse probability of treatment weighting (IPTW) and multivariable Cox regression adjusted for measured covariates, residual confounding from unmeasured variables cannot be ruled out. Specifically, unquantified behavioral parameters—such as baseline personal hygiene practices, individual socioeconomic status, general health-seeking behaviors, or higher psychological distress regarding debris accumulation—may have heavily influenced both treatment choice and clinical outcomes instead of the intervention itself. Furthermore, because baseline bladder parameters like individual bladder capacity, daily compliance, and debris severity or volume were not systematically quantified or graded via standardized ultrasonic or visual scales, the study lacks direct mechanistic insight and cannot establish a definitive dose–response relationship. Consequently, these unmeasured behavioral and physiological factors may have influenced the observed outcomes, meaning these associations cannot be interpreted as definitive proof of therapeutic efficacy. The late Kaplan–Meier curve separation in SCI subgroup must be interpreted with extreme caution due to heavy censoring and minimal data points beyond 24 months. To adequately control for these deep health-seeking and physiological biases, future randomized controlled trials (RCTs) should employ centralized, allocation-concealed randomization stratified by center and baseline catheter material. Additionally, future trial protocols could implement an active-control sham design (e.g., a non-therapeutic rigid or flexible scope insertion without irrigation) coupled with standardized, electronic hygiene diaries to robustly capture and adjust for participant behavioral compliance.

We now note that adverse events within our 26-month window were Clavien–Dindo Grade I (mild self-limiting hematuria or transient discomfort), extended longitudinal tracking is vital to actively screen for insidious structural complications such as localized bladder neck fibrosis, iatrogenic urethral strictures, progressive detrusor wall scarring or procedure refusal due to repetitive discomfort. Adherence to the scheduled cystoscopy protocol was high (91.2%), with only minor scheduling deviations. No patient discontinued participation due to procedure-related discomfort, suggesting acceptable short-term tolerability. However, formal quality-of-life assessments were not performed and should be included in future trials. Additionally, standard urine cultures only detect planktonic pathogens and may miss biofilm-related or anaerobic organisms. Future studies employing next-generation sequencing (NGS) on stored samples could provide deeper insight into microbiome and biofilm dynamics.”

Other limitations include the modest sample size, especially in the SCI control group, single-center setting in Taiwan and relatively short median follow-up of 26 months. The clinical feasibility and long-term tolerability of performing flexible cystoscopy every three months also require further evaluation, as this approach imposes logistical demands on both patients and healthcare systems. Our single-center tertiary care protocol may have limited generalizability to standard community practices or resource-poor settings. While the intervention appears to reduce emergency visits and hospitalizations associated with major UTIs and catheter blockages, formal cost-effectiveness analyses are required. For context, under Taiwan’s National Health Insurance (NHI) system, the reimbursement for a routine outpatient flexible cystoscopy is approximately 1500–1800 NHI points (roughly 56 USD), whereas a single hospitalization for CAUTIs can easily exceed 30,000–45,000 NHI points (approximately 1100–1600 USD) [26]. While the intervention may have favorable cost implications given the high cost of CAUTI-related hospitalizations, no formal health economic analysis was performed. Such evaluation is required before widespread adoption.

## 5. Conclusions

In conclusion, our study demonstrated that proactive, gentle, and regular cystoscopic debris removal reduces catheter blockages, but it failed to significantly reduce symptomatic UTI rates in the overall heterogeneous cohort. The observed reduction in UTIs among patients with SCI should be considered exploratory and hypothesis-generating. Due to the limitations of the self-selected design, potential biases, and modest sample size, the observed associations cannot establish efficacy. Larger, multicenter randomized controlled trials are strongly warranted to confirm efficacy, safety, optimal intervention intervals, cost-effectiveness, and generalizability of this approach.

## Figures and Tables

**Table 1:** Baseline Characteristics Before and After IPTW (Overall Cohort).

Variable	Unweighted Control (*n* = 63)	Unweighted Intervention (*n* = 141)	SMD (Unweighted)	Weighted Control	Weighted Intervention	SMD (Weighted)
Age, years (mean ± SD)	58.2 ± 14.5	56.9 ± 15.0	0.088	57.4 ± 14.7	57.3 ± 14.8	0.007
Male, *n* (%)	47 (74.6)	103 (73.0)	0.037	73.8%	73.6%	0.005
Spinal cord injury, *n* (%)	22 (34.9)	49 (34.8)	0.003	34.9%	34.8%	0.002
Diabetes mellitus, *n* (%)	19 (30.2)	41 (29.1)	0.024	29.4%	29.3%	0.003
Duration of catheterization, years (median, IQR)	3.2 (1.7–5.8)	3.4 (1.9–6.0)	0.071	3.3 (1.8–5.9)	3.3 (1.9–5.8)	0.009
Prior-year UTI episodes (mean ± SD)	2.1 ± 1.4	2.3 ± 1.6	0.133	2.22 ± 1.52	2.21 ± 1.51	0.006
Suprapubic catheter, *n* (%)	12 (19.0)	31 (22.0)	0.074	21.0%	21.1%	0.003
Hypertension, *n* (%)	26 (41.3)	56 (39.7)	0.032	40.2%	40.1%	0.002

IPTW = Inverse Probability of Treatment Weighting; SD = Standard Deviation; *n* = Number of patients; SMD = Standardized Mean Difference; IQR = Interquartile Range; UTI = Urinary Tract Infection. All weighted SMDs < 0.10, indicating excellent balance after IPTW.

**Table 2:** Clinical Outcomes (Overall Cohort).

Outcome	Control (*n* = 63)	Intervention (*n* = 141)	*p*-Value
Catheter blockage requiring intervention, *n* (%)	14 (22.2)	11 (7.8)	0.004 ^a^
Any symptomatic UTI, *n* (%)	31 (49.2)	52 (36.9)	0.092 ^a^
Major UTI (admission/IV antibiotics/fever), *n* (%)	12 (19.0)	14 (9.9)	0.071 ^a^
Median UTI episodes per patient (IQR)	1 (0–2)	0 (0–1)	0.035 ^b^

Crude proportions shown for interpretability. All survival analyses conducted on IPTW-weighted data. ^a^ χ^2^ test; ^b^ Mann–Whitney U test. *n* = Number of patients; UTI = Urinary Tract Infection; IV = Intravenous; IQR = Interquartile Range.

**Table 3:** Procedure-Related Adverse Events (Intervention Group, *n* = 141).

Adverse Event	*n* (%)
Mild hematuria (self-limited)	6 (4.3)
Transient low-grade fever	4 (2.8)
Minor discomfort	7 (5.0)
Any Clavien–Dindo Grade ≥ II	0 (0)
Procedure discontinuation	0 (0)

Adverse event parameters reflect acute safety data collected within 7 days post-procedure over the 26-month follow-up period. *n* = Number of patients.

**Table 4:** Clinical Outcomes in the Overall Cohort and Spinal Cord Injury (SCI) Subgroup.

Outcome	Overall Cohort (*n* = 204)		SCI Subgroup (*n* = 71)	
	Control (*n* = 63)	Intervention (*n* = 141)	Control (*n* = 22)	Intervention (*n* = 49)
Catheter blockage, *n* (%)	14 (22.2)	11 (7.8)	7 (31.8)	6 (12.2)
*p*-value	—	0.004 ^a^	—	0.048 ^a^
Any symptomatic UTI, *n* (%)	31 (49.2)	52 (36.9)	15 (68.2)	22 (44.9)
*p*-value	—	0.092 ^a^	—	0.068 ^a^
Major UTI *, *n* (%)	12 (19.0)	14 (9.9)	8 (36.4)	9 (18.4)
*p*-value	—	0.071 ^a^	—	0.089 ^a^
Median UTI episodes per patient (IQR)	1 (0–2)	0 (0–1)	2 (1–3)	1 (0–2)
*p*-value	—	0.035 ^b^	—	0.021 ^b^
Median follow-up, months (IQR)	26 (18–34)	26 (18–34)	25 (17–33)	27 (19–35)
*p*-value	—	0.41 ^c^	—	0.62 ^c^

* Major UTI: requiring admission, intravenous antibiotics, or fever > 38 °C. Crude proportions shown. ^a^ χ^2^ test; ^b^ Mann–Whitney U test; ^c^ log-rank test. SCI = Spinal Cord Injury; *n* = Number of patients; UTI = Urinary Tract Infection; IQR = Interquartile Range.

**Table 5:** Multivariable Cox Proportional Hazards Regression Model for Symptomatic UTI (SCI Subgroup, *n* = 71).

Variable	Adjusted HR	95% CI	*p*-Value
Intervention (vs. Control)	0.52	0.28–0.96	0.037
Age (per year)	1.02	0.99–1.04	0.189
Male (vs. Female)	0.85	0.41–1.76	0.662
Diabetes mellitus	1.61	0.82–3.17	0.165
Suprapubic catheter (vs. Urethral)	0.74	0.37–1.48	0.394
Prior-year UTI frequency	1.29	1.08–1.54	0.005

HR = Hazard Ratio; CI = Confidence Interval; SCI = Spinal Cord Injury; UTI = Urinary Tract Infection. Models adjusted for age, sex, diabetes mellitus, catheter type, and prior-year UTI frequency. Proportional hazards assumption was satisfied (Schoenfeld residuals test, *p* > 0.05).

**Table 6:** Urine Culture Results from Symptomatic UTI Episodes.

Parameter	Control Group	Intervention Group	*p*-Value
Number of symptomatic UTI episodes with culture	48	71	-
Positive urine culture (>10^5^ CFU/mL), *n* (%)	41 (85.4)	58 (81.7)	0.612
Polymicrobial infection, *n* (%)	12 (29.3)	15 (25.9)	0.703
Most common pathogens			
- *Escherichia coli*	18 (43.9)	22 (37.9)	0.541
- *Klebsiella pneumoniae*	9 (22.0)	11 (19.0)	0.712
- *Pseudomonas aeruginosa*	5 (12.2)	8 (13.8)	0.812
- *Enterococcus* spp.	4 (9.8)	6 (10.3)	0.923
Multidrug-resistant organisms (MDRO), *n* (%)	14 (34.1)	18 (31.0)	0.742

Cultures were available for 119 out of 152 symptomatic UTI episodes (78.3%). *n* = Number of episodes; UTI = Urinary Tract Infection; CFU = Colony Forming Units; spp. = Species; MDRO = multidrug-resistant organisms (resistance to ≥3 antibiotic classes).

## Data Availability

The data presented in this study are available on request from the corresponding author with a reasonable reason.

## Supplementary Materials

The following supporting information can be downloaded at: https://www.mdpi.com/article/10.3390/jcm15135217/s1, Table S1: Multivariable Cox Proportional Hazards Model Diagnostics and Covariate Parameters (SCI Subgroup, *n* = 71); Table S2: Detailed Survival Analysis for Symptomatic UTI-Free Survival (IPTW-Weighted Data); Table S3: Full-Cohort Multivariable Cox Model Including Treatment × SCI Interaction Term; Table S4: Incidence Rate Analysis (Events per 1000 Catheter-Days).

## References

[1] Kelly T., Ai C., Jung M., Yu K. (2024). Catheter-associated urinary tract infections (CAUTIs) and non-CAUTI hospital-onset urinary tract infections: Relative burden, cost, outcomes and related hospital-onset bacteremia and fungemia infections. Infect. Control Hosp. Epidemiol..

[2] Patel P.K., Advani S.D., Kofman A.D., Lo E., Maragakis L.L., Pegues D.A., Pettis A.M., Saint S., Trautner B., Yokoe D.S. (2023). Strategies to prevent catheter-associated urinary tract infections in acute-care hospitals: 2022 Update. Infect. Control Hosp. Epidemiol..

[3] Burton D.C., Edwards J.R., Horan T.C., Jernigan J.A., Fridkin S.K. (2011). Trends in catheter-associated urinary tract infections in adult intensive care units-United States, 1990–2007. Infect. Control Hosp. Epidemiol..

[4] Scruggs-Wodkowski E., Kidder I., Meddings J., Patel P.K. (2024). Urinary Catheter-Associated Infections. Infect. Dis. Clin. N. Am..

[5] Kohler K.N., La Bella A.A., Flores-Mireles A.L. (2026). Host Pathogen Interactions during Catheter-Associated Urinary Tract Infections. ACS Infect. Dis..

[6] Sequi M.B., Lombardo R., Coppola L.M., Rosato E., Romagnoli M., De Cillis S. (2026). Accuracy, readability, and understandability of EAU Guidelines Bot for urinary infections guidelines. Minerva Urol. Nephrol..

[7] Westgeest A.C., van Uhm J.I.M., Pattacini L., Rozemeijer W., Schout B.M.A., Groenwold R.H.H., Geerlings S.E., Lambregts M.M. (2024). Catheter replacement in catheter-associated urinary tract infection: Current state of evidence. Eur. J. Clin. Microbiol. Infect. Dis..

[8] Waites K.B., Canupp K.C., Roper J.F., Camp S.M., Chen Y. (2006). Evaluation of 3 methods of bladder irrigation to treat bacteriuria in persons with neurogenic bladder. J. Spinal Cord Med..

[9] Stevenson S.M., Lau G.A., Andolsek W.C., Presson A.P., Cartwright P.C. (2018). Bladder debris on ultrasound as a predictor for positive urine culture in a pediatric population. J. Pediatr. Urol..

[10] Choi C., Kim D.S., Choi J.B., Choi T., Lee J.W. (2026). Mechanisms and clinical implications of bacterial persistence in recurrent urinary tract infections. Investig. Clin. Urol..

[11] Shepherd A.J., Mackay W.G., Hagen S. (2017). Washout policies in long-term indwelling urinary catheterisation in adults. Cochrane Database Syst. Rev..

[12] Kranz J., Bartoletti R., Bruyère F., Cai T., Geerlings S.E., Köves B., Schubert S., Pilatz A., Veeratterapillay R., Wagenlehner F.M.E. (2024). European Association of Urology Guidelines on Urological Infections: Summary of the 2024 Guidelines. Eur. Urol..

[13] D’Incau S., Atkinson A., Leitner L., Kronenberg A., Kessler T.M., Marschall J. (2023). Bacterial species and antimicrobial resistance differ between catheter and non-catheter-associated urinary tract infections: Data from a national surveillance network. Antimicrob. Steward. Healthc. Epidemiol..

[14] Sartori A.M., Kessler T.M., Castro-Díaz D.M., de Keijzer P., Del Popolo G., Ecclestone H. (2024). Summary of the 2024 Update of the European Association of Urology Guidelines on Neurourology. Eur. Urol..

[15] Antimicrobial Resistance Collaborators (2022). Global burden of bacterial antimicrobial resistance in 2019: A systematic analysis. Lancet.

[16] Garcia M.M., Gulati S., Liepmann D., Stackhouse G.B., Greene K., Stoller M.L. (2007). Traditional Foley drainage systems—Do they drain the bladder?. J. Urol..

[17] Sinclair L., Hagen S., Cross S. (2011). Washout policies in long-term indwelling urinary catheterization in adults: A short version cochrane review. Neurourol. Urodyn..

[18] Werneburg G.T. (2022). Catheter-Associated Urinary Tract Infections: Current Challenges and Future Prospects. Res. Rep. Urol..

[19] Cox L., He C., Bevins J., Clemens J.Q., Stoffel J.T., Cameron A.P. (2017). Gentamicin bladder instillations decrease symptomatic urinary tract infections in neurogenic bladder patients on intermittent catheterization. Can. Urol. Assoc. J..

[20] Lo E., Nicolle L.E., Coffin S.E., Gould C., Maragakis L.L., Meddings J., Pegues D.A., Pettis A.M., Saint S., Yokoe D.S. (2014). Strategies to prevent catheter-associated urinary tract infections in acute care hospitals: 2014 update. Infect. Control Hosp. Epidemiol..

[21] Kwon M., Ahmad A., Lott N., Blatt A. (2026). Intravesical therapy for recurrent urinary tract infection: A systematic review and meta-analysis. BJU Int..

[22] Kobatake K., Inoue S., Takemoto K., Fukushima T., Sekino Y., Ikeda K. (2022). Evaluation of urinary catheters for effective manual bladder washout. Sci. Rep..

[23] Drake M.J., Clavica F., Murphy C., Fader M.J. (2024). Innovating Indwelling Catheter Design to Counteract Urinary Tract Infection. Eur. Urol. Focus.

[24] Pannek J., Wöllner J. (2017). Management of urinary tract infections in patients with neurogenic bladder: Challenges and solutions. Res. Rep. Urol..

[25] Chernev I., Yan K. (2009). Position-dependent urinary retention in a traumatic brain injury patient: A case report. Cases J..

[26] Chen Y.J., Chen F.L., Chen J.H., Wu M.T.M., Chien D.S., Ko Y. (2020). Costs and length of sepsis-related hospitalizations in Taiwan. Medicine.

