# Supplementary material for: Proactive Cystoscopic Debris Removal for Reducing Catheter Blockage in Patients with Long-Term Indwelling Catheters: A Prospective Self-Selected Cohort Study with Exploratory Subgroup Analysis on Urinary Tract Infections
Source: J Clin Med. 2026 Jul 3;15(13):5217. doi: 10.3390/jcm15135217 (PMC13362908; doi:10.3390/jcm15135217)
Supplement: Supplementary file 1 [file jcm-15-05217-s001.zip › jcm-4344929-supplementary.pdf]

Supplementary Table S1 Multivariable Cox Proportional Hazards Model Diagnostics and Covariate Parameters (SCI Subgroup, n=71)

**Covariate Variable**	**B (Coeff.)**	**SE**	**Hazard Ratio (HR)**	**95% CI for HR**	**p-value**	**Schoenfeld Residuals (p-value)**
Intervention (vs. Control)	-0.654	0.312	0.52	0.28 – 0.96	0.037	0.724
Age (per year increments)	0.020	0.012	1.02	0.99 – 1.04	0.189	0.511
Male (vs. Female)	-0.163	0.371	0.85	0.41 – 1.76	0.662	0.819
Diabetes mellitus	0.476	0.345	1.61	0.82 – 3.17	0.165	0.435
Suprapubic catheter (vs. Urethral)	-0.301	0.353	0.74	0.37 – 1.48	0.394	0.602
Prior-year UTI frequency	0.255	0.091	1.29	1.08 – 1.54	0.005	0.783

Supplementary Table S2 Detailed Survival Analysis for Symptomatic UTI-Free Survival (IPTW-Weighted Data)

Overall Cohort (n=204)

**Parameter**	**Control Group**	**Intervention Group**
Total patients (weighted)	63	141
Number of symptomatic UTI events	31	52

Parameter	Control Group	Intervention Group
Number censored	4	9
Median time to first UTI (months)	14.2 (95% CI 9.8–18.6)	19.8 (95% CI 15.4–24.1)
12-month UTI-free survival (%)	68.3	75.9
24-month UTI-free survival (%)	52.4	64.7

SCI Subgroup (n=71)

Parameter	Control (n=22)	Intervention (n=49)
Number of symptomatic UTI events	15	22
Number censored	2	4
Median time to first UTI (months)	11.5 (95% CI 7.2–15.8)	21.3 (95% CI 16.5–26.1)
12-month UTI-free survival (%)	45.5	67.3
24-month UTI-free survival (%)	31.8	55.1

Note: Analyses performed on IPTW-weighted data. Proportional hazards assumption satisfied

(Schoenfeld residuals global test p=0.68). Log-rank test p=0.03 in SCI subgroup.

Supplementary Table S3. Full-Cohort Multivariable Cox Model Including Treatment × SCI Interaction Term

Variable	Adjusted HR	95% CI	p-value
Intervention (vs Control)	0.78	0.48 – 1.27	0.318
SCI (yes vs no)	1.89	1.12 – 3.18	0.017

**Variable**	**Adjusted HR**	**95% CI**	**p-value**
Intervention × SCI Interaction	0.48	0.24 – 0.97	**0.042**
Age (per year)	1.01	0.99 – 1.03	0.214
Male (vs Female)	0.92	0.51 – 1.65	0.778
Diabetes mellitus	1.48	0.89 – 2.46	0.132
Suprapubic catheter (vs Urethral)	0.81	0.47 – 1.39	0.448
Prior-year UTI frequency	1.22	1.08 – 1.38	0.002

Supplementary Table S4. Incidence Rate Analysis (Events per 1000 Catheter-Days)

**Outcome**	**Control Group (Rate)**	**Intervention Group (Rate)**	**Incidence Rate Ratio (95% CI)**	**p-value**
Symptomatic UTI (Overall Cohort)	2.18	1.67	0.77 (0.51 – 1.15)	0.198
Symptomatic UTI (SCI Subgroup)	3.92	2.14	**0.55 (0.31 – 0.96)**	**0.034**
